# Cardioprotective effects of the novel curcumin analogue C66 in diabetic mice is dependent on JNK2 inactivation

**DOI:** 10.1111/jcmm.13924

**Published:** 2018-10-15

**Authors:** Cheng Li, Xiao Miao, Yan Lou, Zhengyang Lu, Binay Kumar Adhikari, Yangwei Wang, Quan Liu, Jian Sun, Yonggang Wang

**Affiliations:** ^1^ Department of Cardiovascular Center The First Hospital of Jilin University Changchun Jilin China; ^2^ The Second Hospital of Jilin University Changchun Jilin China

**Keywords:** apoptosis, C66, curcumin, diabetic cardiomyopathy, JNK2, oxidative stress

## Abstract

**Aim:**

Diabetic cardiomyopathy is an independent cardiac injury that can develop in diabetic individuals. Our previous study showed that C66, a curcumin analogue, protects against diabetes‐induced cardiac damage. The present study sought to reveal the underlying mechanisms of C66‐mediated cardioprotection.

**Methods:**

An experimental diabetic model was established using JNK2^−/−^ and wild‐type (WT) mice. C66 (5 mg/kg) was administered orally every other day for 3 months. Body weight, plasma glucose levels, cardiac function, and structure were measured. Masson trichrome and TUNEL staining were used to assess myocardial fibrosis and apoptosis, respectively. mRNA and protein levels of inflammation, fibrosis, oxidative stress, and apoptosis molecules were measured by quantitative PCR and Western blot, respectively.

**Results:**

Neither C66 treatment nor JNK2 knockout affected body weight or plasma glucose levels. Cardiac inflammation, fibrosis, oxidative stress, and apoptosis were increased in WT diabetic compared to WT control mice, all of which were attenuated by C66 treatment. However, these pathological and molecular changes induced by diabetes were eliminated in JNK2^−/−^ diabetic mice compared to JNK2^−/−^ control mice, and C66 treatment did not further affect these parameters in JNK2^−/−^ diabetic mice.

**Conclusions:**

Our results indicate that C66 ameliorates diabetic cardiomyopathy by inhibiting JNK2 relative pathways.

## 1 INTRODUCTION

As the leading cause of morbidity and mortality in patients with diabetes, diabetic cardiomyopathy imposes enormous burden on individuals and public health. Diabetic cardiomyopathy is defined as ventricular dysfunction in the absence of coronary artery disease, which is complex and can involve enhanced myocardial inflammation, cardiac fibrosis, oxidative stress, and apoptosis.^1^ Potential mechanisms extend beyond consequences of diabetes associated hyperglycaemia and reflect the interaction of multiple factors, which in combination may adversely affect cardiac structure and function. However, the underlying mechanisms that are responsible for diabetic cardiomyopathy development have not been completely elucidated.

Oxidative cellular damage contributes to diabetic cardiomyopathy pathogenesis. C‐Jun N‐terminal kinases (JNKs) are members of the mitogen‐activated protein kinase (MAPK) family and are major signal transducers mediating the physiological response to cellular stressors. Nuclear factor (erythroid‐derived 2)‐like 2 (Nrf2) is a transcription factor that controls redox balance through regulating expression of many antioxidant and anti‐inflammatory genes.^2^ It has been reported that JNK inactivation can significantly activate Nrf2 and subsequently attenuate diabetes‐induced inflammation, fibrosis, and oxidative stress.^3^ Furthermore, JNK2 deficiency can protect the liver from oxidative injury induced by haemorrhage and resuscitation.^4^

Diabetes‐induced oxidative stress can trigger JNK activation, which is considered a critical factor in the pro‐apoptotic signalling pathway.^5^ Foxo3a, a key downstream molecule of JNK activation, can modulate various cellular processes, including cell survival, oxidative stress, and apoptosis.^6^ Other studies have reported that Foxo3a is also regulated by the phosphoinositide 3‐kinase (PI3K)/Akt pathway. Activated Akt phosphorylates and inactivates Foxo3a, traps Foxo3a in the cytoplasm, and impairs its transcriptional activity,^7^ promoting cell survival. In contrast, if Akt is inhibited, Foxo3a can be activated by JNK2 and enter the nucleus with increased transcriptional activity 8 to induce apoptosis. Therefore, Foxo3a‐induced apoptosis may depend on a competitive interaction with JNK2 and PI3K/Akt.

C66, a new curcumin analogue, has anti‐inflammatory, anti‐fibrotic, antioxidative, and anti‐apoptotic properties in diabetic mice, and also significantly suppresses JNK activity.^9^, ^10^ In addition, we found that C66 has a high affinity for JNK2 in rat renal proximal tubular cells under conditions of high glucose, suggesting that C66 may directly target JNK2.^11^ Therefore, we suggested that the protective effects of C66 in diabetes‐induced myocardial injury are mediated by inhibiting JNK2 function. To test this hypothesis, we established a type 1 diabetic model in JNK2 gene knockout (JNK2^−/−^) and wild‐type (WT) mice and investigated whether JNK2 depletion abolishes the beneficial effects of C66 on cardiac injury.

## 2 METHODS

### 2.1 Animal experiments

Male 6‐week‐old JNK2^−/−^ mice on the B6.129S2‐Mapk9tm1Flv/J genetic background and their WT littermates were purchased from the Jackson Laboratory and housed in the Animal Center of Jilin University. All mice were housed under a 12 h/12 h light/dark cycle at 22°C with free access to tap water and standard chow. Wild‐type and JNK2^−/−^ mice were intraperitoneally injected with streptozotocin (STZ) ([150 mg/kg; Sigma‐Aldrich; dissolved in 0.1 M sodium citrate [pH 4.5]) to induce diabetes. Mice in the control group received injections of sodium citrate buffer. Three days after injection, mice with blood glucose levels ≥250 mg/dL were considered diabetic. Diabetic WT and JNK2^−/−^ mice were randomly divided into two groups to receive vehicle or C66 treatment. Each group had eight mice. C66 (dissolved in 1% sodium carboxymethyl cellulose solution) was administered orally to mice at 5 mg/kg every other day for 3 months. The vehicle groups were treated with 1% sodium carboxymethyl cellulose solution. Body weight was measured biweekly.

### 2.2 Blood glucose and echocardiography

Plasma glucose levels were measured using a glucometer (Accu‐Chek; Roche, Basel, Switzerland) at 8:00 am every 2 weeks. At 68 days after STZ injection, transthoracic echocardiography was performed under general anaesthesia using a small animal echo system (Vevo 770; Visual Sonics, Toronto, Canada) with a high‐frequency ultrasound probe (RMV‐707B). Left ventricular (LV) ejection fraction (EF), LV fractional shortening (FS), LV end‐systolic diameter (LVIDs), LV end‐diastolic diameter (LVIDd), LV posterior wall end‐systolic thickness (LVPWs), and LV posterior wall end‐diastolic thickness (LVPWd) were calculated. And the left ventricular mass index (LV mass I) was calculated. Animals were killed via ether anaesthesia and cervical dislocation, and blood and tissues were collected and stored appropriately for future analyses.

### 2.3 Histology

Hearts were fixed in 4% paraformaldehyde, paraffin‐embedded, and cut into 4‐μm‐thick sections. The sections were deparaffinized and stained with haematoxylin and eosin and Masson's trichrome to determine morphology and myocardial fibrosis, respectively.

### 2.4 Real‐time quantitative PCR

Total RNA was extracted from heart tissues using the AxyPrep™ multisource total RNA kit (Axygen Scientific, Inc., Hangzhou, China). RNA was reverse transcribed to cDNA using the TransScript All‐in‐One first‐strand cDNA synthesis SuperMix (Transgen Biotech, Inc., Beijing, China). Real‐time PCR was performed using the TransStart Top Green qPCR SuperMix (Transgen Biotech, Inc.) and the ABI 7300 Real‐Time qPCR system. All PCR experiments were performed in triplicate. The primers were purchased from Sangon Biotech (Shanghai, China) and the sequences are listed in Table 1 **.**

**Table 1:** Primer sequences for real‐time quantitative PCR

Gene	Forward primer	Reverse primer
CTGF	GGGCCTCTTCTGCGATTTC	ATCCAGGCAAGTGCATTGGTA
TGF‐B1	CTCCCGTGGCTTCTAGTGC	GCCTTAGTTTGGACAGGATCTG
ICAM‐1	GTGATGCTCAGGTATCCATCCA	CACAGTTCTCAAAGCACAGCG
MCP‐1	TTAAAAACCTGGATCGGAACCAA	GCATTAGCTTCAGATTTACGGGT
HO‐1	AAGCCGAGAATGCTGAGTTCA	GCCGTGTAGATATGGTACAAGGA
SOD‐1	AACCAGTTGTGTTGTCAGGAC	CCACCATGTTTCTTAGAGTGAGG

### 2.5 Western blot

Homogenized heart tissues were lysed and protein concentrations were measured using the Enhanced BCA Protein Assay Kit (Beyotime, Inc., Shanghai, China) . Lysates were separated by SDS‐PAGE and then transferred onto 0.45 μm polyvinyldene difluoride membranes. Each membrane was pre‐incubated in tris‐buffered saline (TBS) containing 5% non‐fat milk for 1 hour at room temperature and then incubated with specific primary antibodies overnight at 4°C. After three washes in TBS with Tween 20, the membranes were incubated with secondary antibodies conjugated to horseradish peroxidase, and immunoreactive bands were visualized using the enhanced chemiluminescence reagent (Immobilon ECL Ultra Western HRP Substrate; Millipore Sigma, Inc., Billerica, MA, USA). Image J was used to analyse protein band density.

### 2.6 Assessment of lipid peroxidation

Lipid peroxidation was estimated by measuring the peroxidation products malondialdehyde (MDA) and 4‐hydroxynonenal‐His adducts (4‐HNE). Malondialdehyde was measured in homogenized heart tissues using a commercially available kit (Nanjing Jiancheng Bioengineering Institute, China). Each sample was tested twice. Malondialdehyde levels were expressed in nanomoles per milligram tissue. 4‐hydroxynonenal‐His adducts and three antioxidant enzymes, including heme oxygenase‐1 (HO‐1), NADPH quinone oxidoreductase (NQO‐1), and superoxide dismutase‐1 (SOD‐1), were measured by Western blot.

### 2.7 TUNEL assay

Tissues were fixed with formaldehyde, and antigen retrieval was performed by heat mediated citrate buffer, pH6. The tissues were incubated with 0.5% tritonX‐100 for 15 minutes at room temperature, and 1% BSA for 30 minutes at 37°C. The tissues were incubated with sarcomeric α‐actinin antibody at 1/200 dilution for 16 hours at 4°C. Secondary antibody was used at 1: 350 (coloured red). Apoptosis was measured using a TUNEL staining kit (DeadEnd^TM^ Colorimetric TUNEL System; Promega, Inc., Madison, WI, USA) according to the manufacturer's instructions. Briefly, paraffin sections were dewaxed and incubated with TdT and fluorescein‐labelled dUTP for 60 minutes at 37°C. Nuclei were counterstained with DAPI. Images were acquired using confocal microscopy. Results were analysed using Image J. All assays and analyses were performed in a blind manner.

### 2.8 Statistical analysis

Data are presented as means ± SD for each group. Comparisons among groups were performed using one‐way ANOVA with post hoc pairwise repetitive comparisons using Tukey test with Origin 8.0 Lab data analysis and GraphPad Prism 6.0 graphing software. Statistical significance was considered as *P* < 0.05.

## 3 RESULTS

### 3.1 Body weight, blood glucose, and heart weight

**Figure 1:**
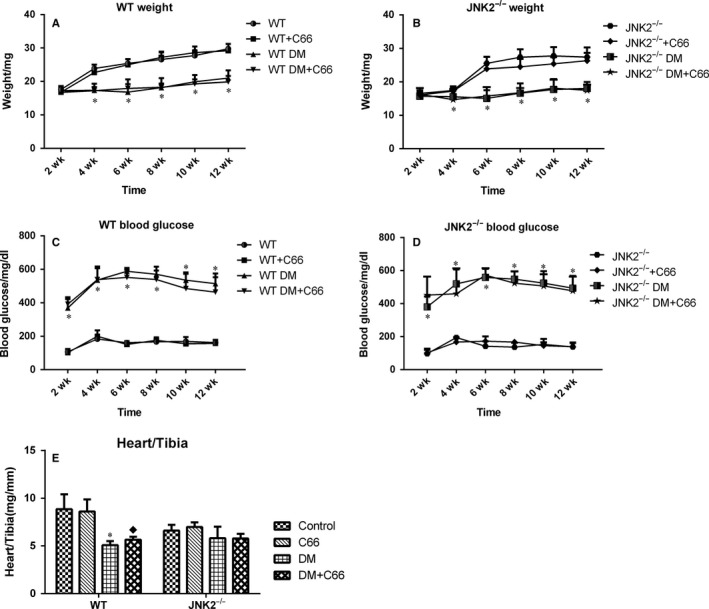
Metabolic profiles of diabetic mice. Body weight (A,B) and blood glucose levels (C,D) were measured biweekly. (E) Heart weight to tibia length ratios were measured at the end of the study. Data are presented as means ± SD (n = 8). *P < 0.05 DM vs corresponding control groups, ^◆^ *P* < 0.05 DM+C66 vs corresponding DM groups. DM, diabetes mellitus

Body weights of both WT (Figure 1A) and JNK2^−/−^ (Figure 1B) diabetic mice were significantly decreased by the fourth week. Blood glucose levels of both WT (Figure 1C) and JNK2^−/−^ (Figure 1D) diabetic mice were significantly increased by the second week. Body weights and blood glucose levels were similar between WT and JNK2^−/−^ diabetic mice following C66 treatment, suggesting that C66 does not affect weight gain and glycaemic control. At 3 months, heart weight to tibia length ratios were lower in the WT diabetic mice compared to the WT control group (*P* < 0.05), and C66 treatment slightly increased this ratio (Figure 1E). In contrast, there was no difference in the heart weight to tibia length ratios between the diabetic and control JNK2^−/−^ mice, and C66 treatment did not alter the ratio in either group (Figure 1E).

### 3.2 Cardiac remodelling and dysfunction

**Figure 2:**
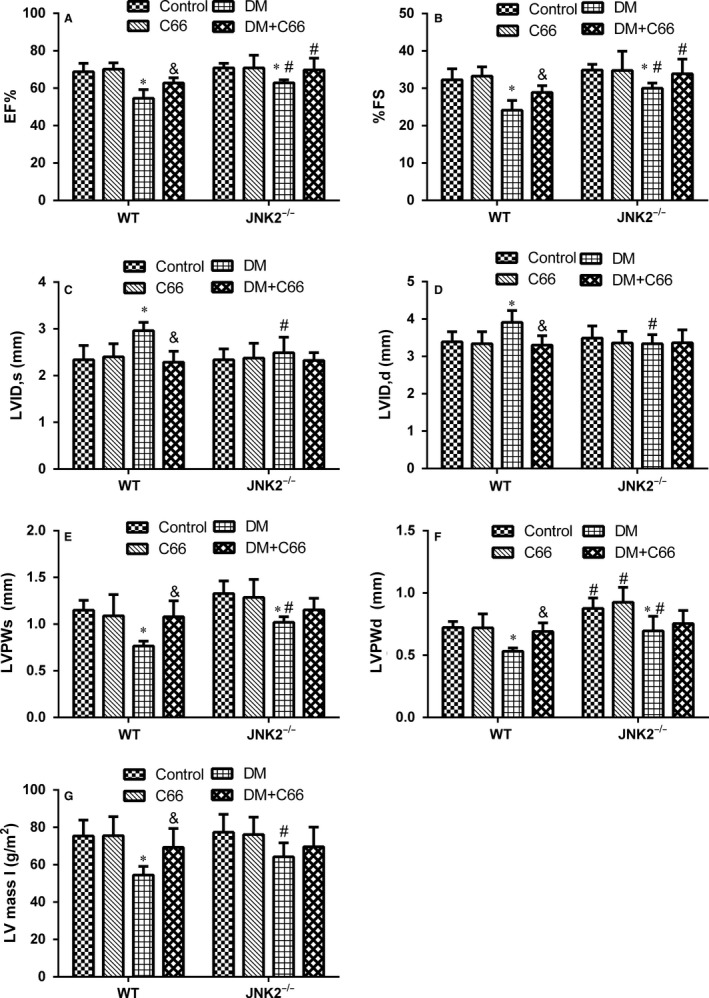
Effects of C66 on diabetes‐induced cardiac remodelling and dysfunction in WT and JNK2^−/−^ mice. Cardiac functional (A,B) and structural (C‐F) changes were evaluated by transthoracic echocardiography. EF, ejection fraction; FS, fractional shortening; LVIDs, left ventricular end‐systolic diameter; LVIDd, left ventricular end‐diastolic diameter; LVPWs, left ventricular posterior wall end‐systolic thickness; LVPWd, left ventricular posterior wall end‐diastolic thickness. LV mass I, left ventricular mass index. Data are presented as means ± SD (n = 8). *P < 0.05 DM vs corresponding control groups; ^&^ *P* < 0.05 DM+C66 vs corresponding DM groups; ^#^ *P* < 0.05 JNK2^−/−^ mice vs corresponding WT mice. DM, diabetes mellitus

Cardiac structure and function were evaluated by transthoracic echocardiography (Figure 2A‐F). The systolic functional parameters, EF and FS, were significantly lower in WT diabetic mice compared to WT control mice (both *P* < 0.05, Figure 2A,B). C66 treatment significantly increased EF and FS in WT diabetic mice (both *P* < 0.05, Figure 2A,B). EF and FS were slightly decreased in the JNK2^−/−^ diabetic group compared to the JNK2^−/−^ control ones (both *P* < 0.05), which were not significantly improved by C66 treatment (Figúre 2A,B). LVIDs and LVIDd were significantly increased, while LVPWs and LVPWd were significantly decreased, in the WT diabetic group compared to the WT control group (all *P* < 0.05, Figure 2C‐F). C66 treatment significantly recovered these indicators (all *P* < 0.05, Figure 2C‐F). LVIDs and LVIDd were not different between the diabetic and control JNK2^−/−^ groups (Figure 2C,D). LVPWs and LVPWd were decreased in the JNK2^−/−^ diabetic group compared to the JNK2^−/−^ control group (both *P* < 0.05, Figure 2E,F). However, C66 treatment did not improve these indicators in the JNK2^−/−^ diabetic group. Interestingly, LVIDs and LVIDd were significantly decreased, while LVPWs and LVPWd were increased, in the diabetic JNK2^−/−^ group compared to the WT diabetic group (all *P* < 0.05, Figure 2C‐F). In addition, we calculated LV mass I, which was significantly decreased in the WT diabetic group compared to the WT control group (*P* < 0.05, Figure 2G); and there was no significant difference among JNK2^−/−^ groups. These results suggest that the inhibition of JNK2 function affects diabetic cardiomyopathy development and that the cardioprotective effects of C66 are mediated by JNK2.

### 3.3 Phosphorylation of JNK

**Figure 3:**
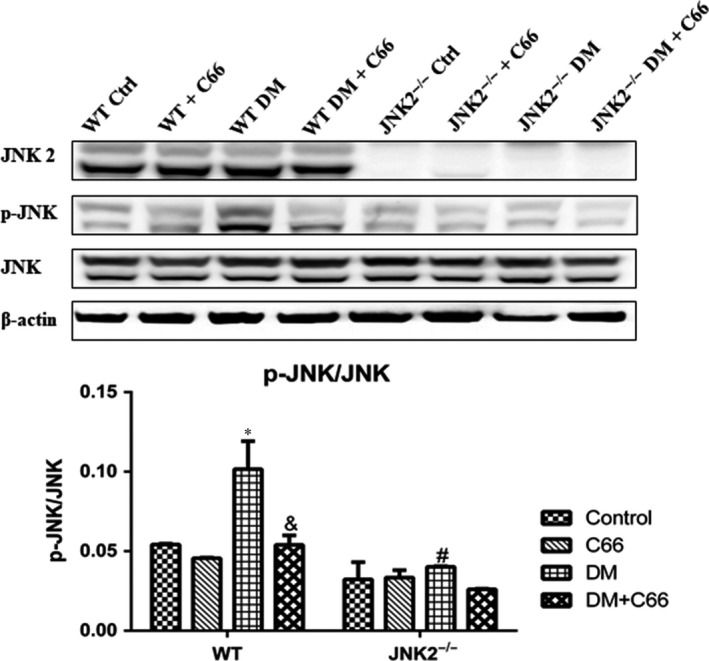
Effects of C66 on cardiac p‐JNK/JNK expression. p‐JNK/JNK expression was analysed by Western blot. n = 8, *P < 0.05 DM vs corresponding control group; ^&^ *P* < 0.05 DM+C66 vs corresponding DM; ^#^ *P *< 0.05 JNK2^−/−^ mice vs corresponding WT mice. DM, diabetes mellitus

The phosphorylation levels of JNK are representative of its activity. Phosphorylated (p)‐JNK was significantly reduced in the hearts of WT diabetic mice compared to the WT control mice (*P* < 0.05, Figure 3), and this affect was reversed by C66 treatment (*P* < 0.05, Figure 3), indicating that C66 can inhibit JNK activity. However, there was no significant change in p‐JNK between JNK2^−/−^ diabetic and control mice (Figure 3). C66 treatment did not affect p‐JNK levels in diabetic JNK2^−/−^ mice (Figure 3). These results indicate that JNK knockout decreases JNK activity in the hearts of diabetic mice and that C66 inhibits diabetes‐induced p‐JNK through inactivating JNK2.

### 3.4 Cardiac fibrosis and inflammation

**Figure 4:**
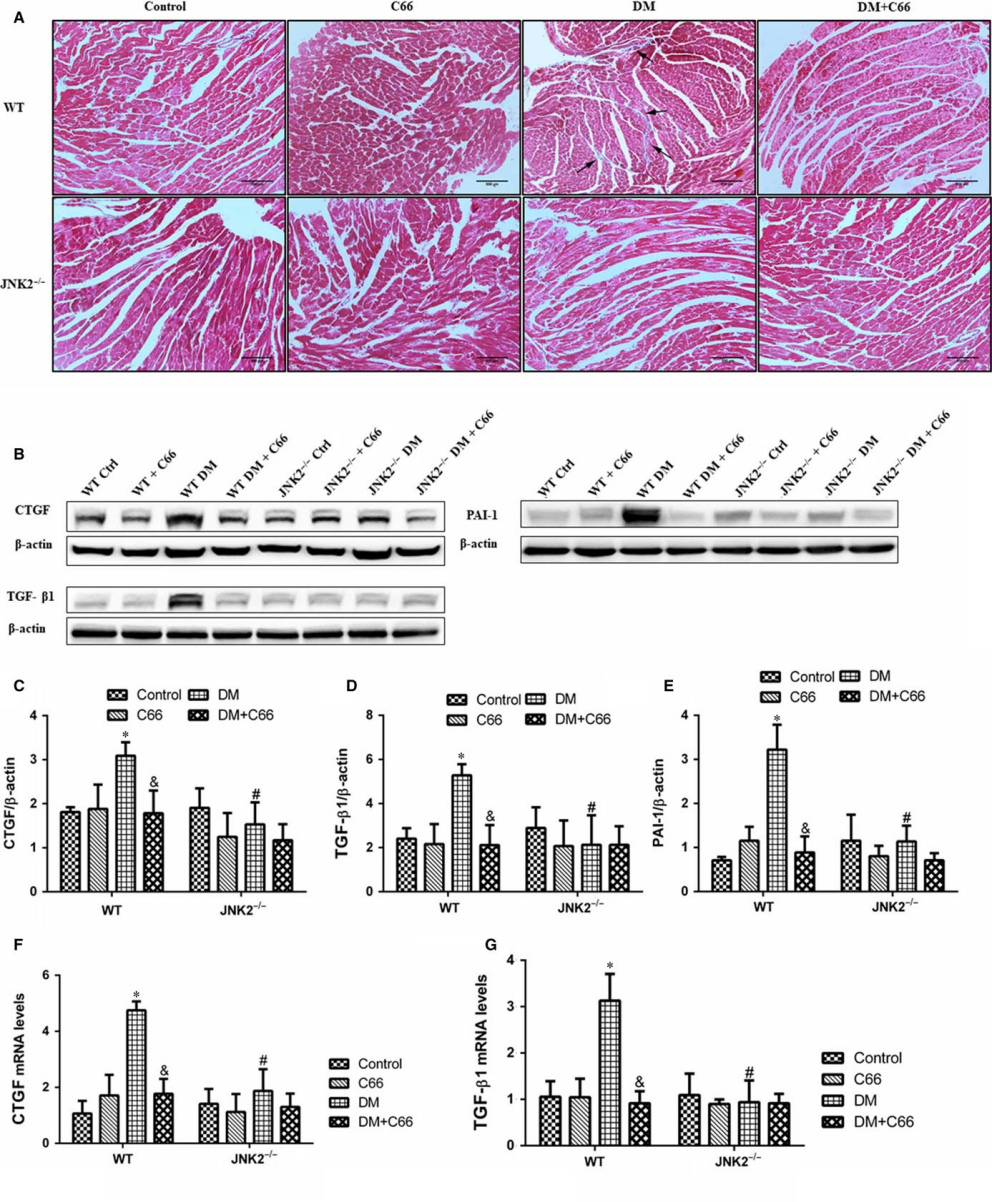
Effects of C66 on diabetes‐induced cardiac fibrosis in WT and JNK2^−/−^ mice. A, Masson's trichrome staining was performed in cardiac tissue (magnification, ×400; scale bar, 50 μm). B and C, Protein expression levels of fibrosis‐associated factors, including CTGF, TGF‐β1, and PAI‐1, were analysed by Western blot. F and G, mRNA expression levels of CTGF and TGF‐β1 were analysed by qPCR. CTGF, connective tissue growth factor; TGF‐β1, transforming growth factor beta 1; PAI‐1, plasminogen activator inhibitor‐1. n = 8, *P < 0.05 DM vs corresponding control group; ^&^ *P* < 0.05 DM+C66 vs corresponding DM; ^#^ *P* < 0.05 JNK2^−/−^ mice vs corresponding WT mice. DM, diabetes mellitus

Diabetes‐induced cardiac fibrosis were shown via Masson's trichrome staining (Figure 4A), and fibrotic markers, including connective tissue growth factor (CTGF), transforming growth factor beta 1 (TGF‐β1), and plasminogen activator inhibitor‐1 (PAI‐1) were measured by Western blot (Figure 4B‐E). mRNA expression levels of CTGF and TGF‐ β1 were measured by qPCR (Figure 4F,G). Light microscopy indicated that there was an obvious amount of collagen identified in interstitial areas in the WT diabetic mice. Treatment with C66 decreased the amount of diabetes‐induced collagen accumulation (Figure 4A). In addition, no obvious fibrosis was observed in the JNK2^−/−^ diabetic groups treated with or without C66 (Figure 4A). CTGF, TGF‐β1, and PAI‐1 expression in the heart were significantly increased in the WT diabetic group compared to the WT control group (all *P* < 0.05), all of which were diminished following C66 treatment (all *P* < 0.05, Figure 4B‐E). CTGF, TGF‐β1, and PAI‐1 expression in the hearts were not different among the four groups of JNK2^−/−^ mice (Figure 4B‐E). Furthermore, CTGF, TGF‐β1, and PAI‐1 expression levels in the JNK2^−/−^ diabetic group were markedly reduced compared to the WT diabetic group (all *P* < 0.05, Figure 4B‐E). Similar results were obtained with qPCR (Figure 4F,G). These findings suggest that C66‐mediated inhibition of JNK2 activity attenuates cardiac fibrosis in diabetic mice.

**Figure 5:**
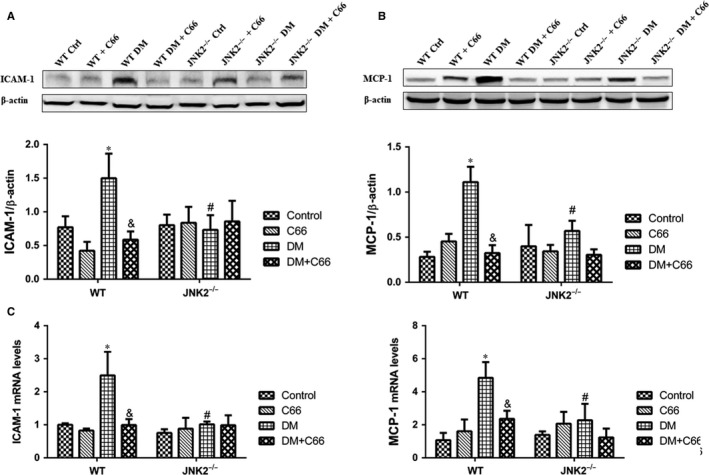
Effects of C66 on diabetes‐induced cardiac inflammation in WT and JNK2^−/−^ mice. Inflammatory factors, ICAM‐1 and MCP‐1, were analysed at the protein level by Western blot (A,B) and the mRNA level by qPCR (C,D). ICAM‐1, intercellular adhesion molecule‐1; MCP‐1, monocyte chemoattractant protein‐1. n = 8, *P < 0.05 DM vs corresponding control group; ^&^ *P* < 0.05 DM+C66 vs corresponding DM; ^#^ *P* < 0.05 JNK2^−/−^ mice vs corresponding WT mice. DM, diabetes mellitus

Protein expression and mRNA levels of inflammatory makers, intercellular adhesion molecule 1 (ICAM‐1) and monocyte chemoattractant protein‐1 (MCP‐1), were measured by Western blot (Figure 5A,B) and qPCR (Figure 5C,D), respectively. ICAM‐1 and MCP‐1 were up‐regulated in the hearts of WT diabetic mice compared to WT control mice, which were reversed by C66 treatment (all *P* < 0.05, Figure 5A‐D). There were no significant differences in ICAM‐1 and MCP‐1 protein expression and mRNA levels among the four groups of JNK2^−/−^ mice (Figure 5A‐D). Interestingly, the expression of these two markers was obviously decreased in JNK2^−/−^ diabetic mice compared to WT diabetic mice (all *P* < 0.05, Figure 5A‐D). These results indicate that C66‐mediated inhibition of JNK2 activity alleviates cardiac inflammation in diabetic mice.

### 3.5 Cardiac oxidative stress

**Figure 6:**
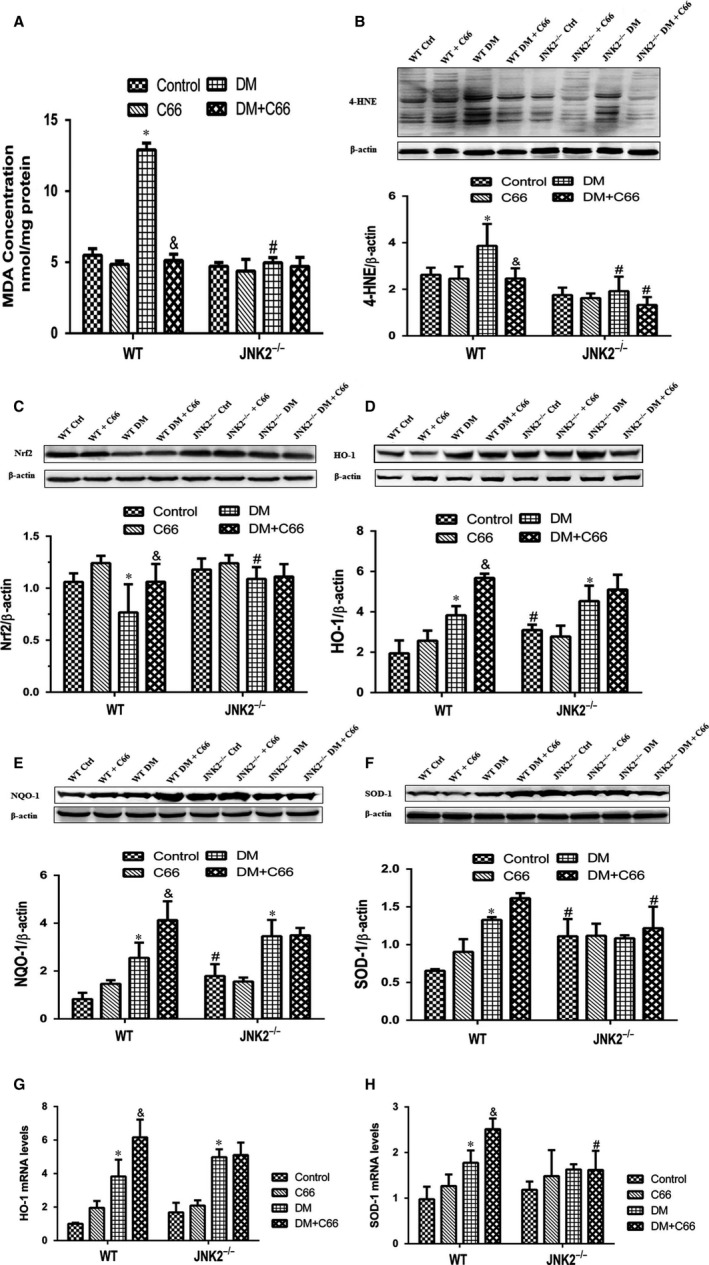
Effects of C66 on diabetes‐induced cardiac oxidative stress in WT and JNK2^−/−^ mice. A, MDA was expressed as nmol/mg protein. B, 4‐HNE was detected by Western blot. C, Nrf2 expression was analysed by Western blot. D‐F, Antioxidative enzymes, including HO‐1, NQO‐1, and SOD‐1, were analysed by Western blot. G and H, mRNA levels of HO‐1 and SOD‐1. MDA, malondialdehyde; 4‐HNE, 4‐hydroxynonenal‐His adducts; Nrf2, nuclear factor (erythroid‐derived 2)‐like 2; HO‐1, heme oxygenase‐1; NQO‐1, NADPH quinone oxidoreductase; SOD‐1, superoxide dismutase‐1. n = 8, *P < 0.05 DM vs corresponding control group, ^&^ *P* < 0.05 DM+C66 vs corresponding DM; ^#^ *P* < 0.05 JNK2^−/−^ mice vs corresponding WT mice. DM, diabetes mellitus

Reactive oxygen species (ROS) production is increased in diabetes, resulting in enhanced oxidative stress, lipid peroxidation, and protein oxidization, which contribute to diabetic cardiomyopathy pathogenesis. We found that MDA cardiac levels were significantly higher in the WT diabetic mice compared to the WT control mice, which were reversed by C66 treatment (*P* < 0.05, Figure 6A). These changes were not observed in JNK2^−/−^ mice (Figure 6A). A significant reduction in MDA was observed in the JNK2^−/−^ diabetic mice compared to the WT diabetic mice (*P* < 0.05, Figure 6A). Similarly, 4‐HNE was significantly increased in the WT diabetic mice compared to the WT control mice, which was prevented by C66 treatment (Figure 6B). No significant difference was observed among the four groups of JNK2^−/−^ mice. The 4‐HNE level was lower in the JNK2^−/−^ diabetic mice compared to the WT diabetic mice (Figure 6B).

To measure Nrf2 expression and its downstream antioxidant genes, protein expression and mRNA levels of Nrf2, HO‐1, NQO‐1, and SOD‐1 were detected by Western blot and qPCR, respectively. Nrf2 was significantly down‐regulated, while HO‐1, NQO‐1 and SOD‐1 were slightly up‐regulated, in the WT diabetic mice compared to WT control mice, all of which were increased after C66 treatment (all *P* < 0.05, Figure 6C‐H). There were no significant difference in the expression of these four molecules between JNK2^−/−^ diabetic mice and JNK2^−/−^ mice administered C66 (Figure 6C‐H).

### 3.6 Cardiomyocyte apoptosis

**Figure 7:**
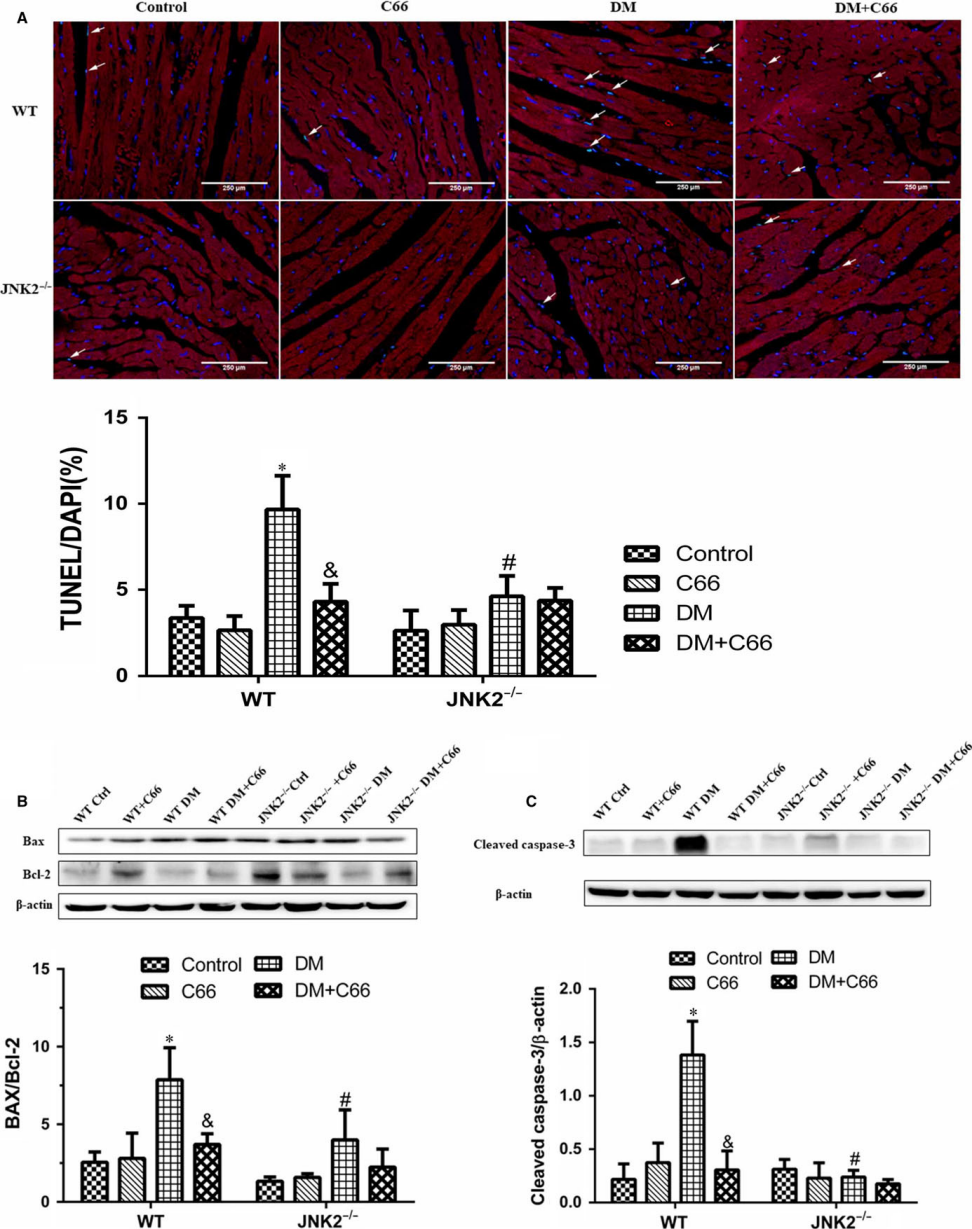
Effects of C66 on diabetes‐induced cardiac apoptosis in WT and JNK2^−/−^ mice. A, TUNEL assay was performed to evaluate apoptosis in cardiomyocytes (green). (cardiomyocytes were marked by sarcomeric α‐actinin staining in red and nuclei were counterstained with DAPI). B, BAX, Bcl‐2, and cleaved caspase‐3 expression levels were detected by Western blot. n = 8, *P < 0.05 DM vs corresponding control groups; ^&^ *P* < 0.05 DM+C66 vs corresponding DM; ^#^ *P* < 0.05 JNK2^−/−^ mice vs corresponding WT mice. DM, diabetes mellitus

We used the TUNEL assay to analyse apoptosis of myocardial cells (cardiomyocytes were marked by sarcomeric α‐actinin staining in red) and Western blot to measure expression of apoptosis‐related proteins, including cleaved caspase‐3, Bax, and Bcl‐2. TUNEL staining showed that there were more apoptotic cells in the WT diabetic mice compared to the WT control mice, and C66 treatment decreased apoptosis (both *P* < 0.05, Figure 7A). Apoptosis was neither abundant nor different among the four JNK2^−/−^ groups (Figure 7A). Interestingly, the hearts from the JNK2^−/−^ diabetic mice had significantly less apoptotic cells compared to the WT diabetic mice (*P* < 0.05, Figure 7A). Similar results were obtained for cleaved caspase‐3, Bax, and Bcl‐2 (Figure 7B).

### 3.7 PI3K/Akt/Foxo3a survival pathway

**Figure 8:**
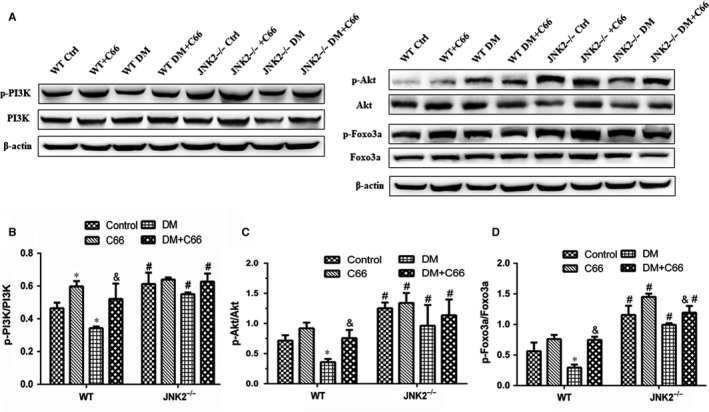
Effects of C66 on activation of the PI3K/Akt/Foxo3a survival pathway in diabetic WT and JNK2^−/−^ mice. p‐PI3K/PI3K, p‐Akt/Akt, and p‐Foxo3a/Foxo3a expression levels were analysed by Western blot. n = 8, *P < 0.05 DM vs corresponding control group; ^&^ *P* < 0.05 DM+C66 vs corresponding DM; ^#^ *P* < 0.05 JNK2^−/−^ mice vs corresponding WT mice. DM, diabetes mellitus

To investigate whether C66 affects activation of the PI3K/Akt/Foxo3a pathway, we measured p‐PI3K, p‐Akt, and p‐Foxo3a in heart tissue by Western blot. As depicted in Figure 8A‐D, we found that diabetes down‐regulated phosphorylation of all three proteins in WT mice, which was reversed by C66 treatment (all *P* < 0.05). However, these changes were not observed in the JNK2^−/−^ groups. In addition, there were significant increases in p‐PI3K, p‐Akt, and p‐Foxo3a in the JNK2^−/−^ diabetic mice compared to the WT diabetic mice (all *P* < 0.05).

## 4 DISCUSSION

The present study demonstrates that the protective effect of C66 in diabetes‐induced cardiac injury is mediated by inhibiting JNK2 activity. Our data show that diabetes‐induced cardiac inflammation, fibrosis, oxidative damage, and apoptosis can be prevented by C66 treatment and that JNK2 deficiency produces similar effects. These results suggest that C66 exerts its cardioprotective effects by inhibiting JNK2 function and activating the Akt/Foxo3a survival pathway.

This study defined the effects of JNK2 function inhibition on diabetes‐induced myocardial damage. Specifically, we found that C66 can prevent increased phosphorylated JNK in WT diabetic mice. However, phosphorylated JNK was not significantly increased in JNK2^−/−^ diabetic mice, and therefore C66 treatment has no effect on JNK2^−/−^ diabetic mice. In addition, C66 treatment prevented diabetes‐induced myocardial remodelling, fibrosis, inflammation, oxidative damage, and apoptosis in WT but not JNK2^−/−^ diabetic mice. These results indicate that C66 protects the heart from diabetic cardiomyopathy via targeting JNK2.

Our previous studies demonstrated that C66 inhibits inflammation, fibrosis, and oxidative stress, which is accompanied by JNK activity inhibition.^10^, ^11^ Evidence has shown that JNK2 activation increases metabolism‐related inflammation, fibrosis, and oxidative stress.^12^, ^13^, ^14^ Consistent with these findings, our present study demonstrated that both C66 treatment and JNK2 knockout markedly reduced diabetic‐induced cardiac inflammation, fibrosis, and oxidative stress; however, C66 had no effect on JNK2^−/−^ diabetic mice. Thus, our data demonstrate that inhibition of JNK2 activity can protect against diabetes‐induced myocardial injury, and C66 protects the heart from diabetic cardiomyopathy by inhibiting JNK2 activity.

Oxidative stress is a key mechanism by which diabetes induces diabetic cardiomyopathy. High glucose concentrations cause an imbalance between production and elimination of ROS, resulting in enhanced oxidative stress, which is accompanied by cardiac inflammation and fibrosis.^15^ JNK is a stress‐activated protein kinase that plays a key role in regulating inflammation, oxidative stress, and apoptosis. JNK2 deletion can decrease oxidative stress.^4^ The present study showed that oxidative stress was induced by diabetes in WT mice and attenuated by C66. However, C66 was not able to suppress the oxidative stress in JNK2^−/−^ diabetic mice, indicating that the anti‐oxidative effects of C66 are dependent on JNK2 activity inhibition.

Nuclear factor (erythroid‐derived 2)‐like 2 regulates cellular detoxification and redox status by inducing expression of multiple antioxidant genes. Evidence has shown that diabetes‐induced oxidative stress is related to JNK activation and Nrf2 inhibition, and suppression of JNK activates Nrf2.^3^ Consistent with the literature, we demonstrated that both C66 treatment and JNK2 deletion activated Nrf2. Moreover, we measured the expression of Nrf2 downstream genes, including HO‐1, NQO‐1, and SOD‐1. Our results show that these antioxidant enzymes were slightly increased in WT diabetic mice, which were probably due to a self‐stress response. C66 treatment markedly increased antioxidant enzyme expression. Similarly, antioxidant enzymes were not significantly changed among JNK2^−/−^ groups. The results from our present study indicate that Nrf2 mediates the antioxidant effects of C66, which are mainly dependent on JNK2.

Foxo3a is a vital downstream effector of the apoptosis signalling cascade. JNK activation has been reported to be involved in Foxo3a‐dependent apoptosis via promoting Foxo3a nuclear translocation and inducing pro‐apoptotic gene expression. However, Foxo3a activation is also regulated by the PI3K/Akt signalling pathway. The forkhead box class O (Foxo) subfamily of transcription factors, which are the downstream targets of the PI3K/Akt signalling cascade, are required for the conduction of various cellular processes, including cell cycle regulation, apoptosis, development, differentiation, invasion, metabolism, migration, oxidative stress, and DNA damage.^16^, ^17^, ^18^ It has been reported that high glucose can inhibit p‐Akt and suppress nuclear translocation of Foxo3a, promoting Foxo3a‐dependent apoptosis in diabetic cardiomyopathy.^19^ In addition, Foxo3a is regulated by JNK, which has been shown to induce Foxo3a activity and nuclear localization by repressing p‐Akt.^20^ Moreover, Davila et al reported that JNK2 is necessary for Foxo3a activation.^8^ We speculated that Foxo3a activation was competitively regulated by both JNK signalling (most likely by JNK2) and PI3K/Akt signalling pathways, both of which have been shown to be down‐regulated in diabetes. The crosstalk between these signalling cascades and the inactivation of Foxo3a may be crucial factors in cardiac apoptosis. Diabetes downregulates the phosphorylation of PI3K, Akt, and Foxo3a, all of which were reversed by C66 in WT mice, but not in JNK2^‐/‐^ mice, indicating that C66 activation of these enzymes is mediated by JNK2 activity inhibition. JNK2 thus competes with PI3K/Akt to regulate the Foxo3a‐depending survival pathway.

In summary, we found that the cardioprotective effects of C66 are dependent on JNK2. C66 protects against diabetic cardiomyopathy via inhibiting JNK2 activation, resulting in decreased cardiac inflammation, fibrosis, oxidative stress, and apoptosis. Furthermore, we found an interaction between the JNK and PI3K/Akt pathways in regulating the Foxo3a‐dependent survival. These results will be valuable for developing strategies to prevent and treat diabetic cardiomyopathy.

## CONFLICT OF INTEREST

No conflicts of interest, financial or otherwise, are declared by the author(s).
